# Synchronous primary breast invasive carcinoma and breast metastasis from high-grade serous ovarian carcinoma: a case report and literature review

**DOI:** 10.3389/fonc.2026.1790056

**Published:** 2026-06-17

**Authors:** Jingge Miao, Nianqiu Liu, Fuhong Gong, Jiayi Chen, Chenxi Wang, Mengping Lin, Na Li, Yilin Chen, Fei Ge, Wenlin Chen

**Affiliations:** 1 Yunnan Key Laboratory of Breast Cancer Precision Medicine, Department of Breast Surgery, The Third Affiliated Hospital of Kunming Medical University, Yunnan Cancer Hospital, Peking University Cancer Hospital Yunnan, Kunming, Yunnan, China; 2 Department of Breast Surgery, The First Affiliated Hospital of Kunming Medical University, Kunming, Yunnan, China

**Keywords:** breast cancer, metastasis, ovarian cancer, tumor, women’s health

## Abstract

**Background:**

Breast metastasis from high-grade serous ovarian carcinoma is rare, and its coexistence with a synchronous primary breast carcinoma is exceptionally uncommon. Distinguishing metastatic ovarian carcinoma from primary breast carcinoma is clinically important because treatment strategies and prognosis differ substantially.

**Case presentation:**

We report a 48-year-old premenopausal woman who presented with pleural effusion, abdominal distension, bilateral adnexal masses, multiple breast nodules, and systemic lymphadenopathy. Pathological examination of the pelvic lesion supported high-grade serous carcinoma, whereas breast biopsy demonstrated two morphologically distinct adenocarcinoma components. Immunohistochemical profiling, including breast-lineage markers and Müllerian/ovarian markers, supported the coexistence of primary breast invasive carcinoma and metastatic ovarian carcinoma involving the breast.

**Intervention and outcome:**

The patient was diagnosed with FIGO stage IVB high-grade serous ovarian carcinoma with breast, pleural, peritoneal, and lymph node involvement, synchronous with primary breast carcinoma. She received neoadjuvant paclitaxel plus carboplatin and achieved a partial response, followed by interval cytoreductive surgery, additional chemotherapy combined with bevacizumab, breast tumor excision, and maintenance therapy with fluzoparib plus bevacizumab. At the last follow-up on December 20, 2025, no evidence of disease progression was observed.

**Conclusion:**

This case highlights the diagnostic difficulty of breast lesions in patients with ovarian carcinoma. Morphology combined with an appropriate immunohistochemical panel is essential for distinguishing primary breast carcinoma from metastatic ovarian carcinoma. Multidisciplinary evaluation and genetic assessment should be considered in similar cases.

## Introduction

Breast cancer is the most common malignancy among women worldwide and remains the leading cause of cancer-related mortality in this population (1). While primary breast cancers predominate, metastatic breast tumors are rare and typically originate from contralateral breast lesions. Metastases to the breast from extramammary solid tumors account for only 0.2%–1.1% of all cases (2).

Ovarian cancer—though not the most prevalent gynecological malignancy—is characterized by high aggressiveness, invasiveness, and recurrence rates. It carries the highest case-fatality rate among female reproductive tract cancers (3, 4), largely due to late detection when distant metastases are already present. Common metastatic sites include the liver (37.5%), lymph nodes (29.4%), lung (28.4%), bone (3.7%), and brain (1%) (5). In contrast, metastasis to the breast from ovarian cancer is exceedingly rare; such cases constitute just 0.03%–0.6% of all malignant breast neoplasms (6).

The coexistence of primary breast carcinoma and ovarian carcinoma metastasis to the breast creates a major diagnostic challenge. We present this case to highlight the value of integrating morphology, immunohistochemistry, imaging, and multidisciplinary assessment.

## Case presentation

### Patient information and clinical findings

A 48-year-old premenopausal woman presented with pleural effusion in February 2024. She had a 16-year history of smoking and no family history of breast or ovarian cancer. Initial thoracoscopic pleurectomy and pleural biopsy at an outside hospital revealed metastatic high-grade serous carcinoma (HGSC).

On admission to our hospital in April 2024, physical examination revealed a firm mass in the left breast (~1.5 × 1.0 cm) and palpable bilateral axillary lymph nodes. Gynecologic examination revealed bilateral adnexal masses (left 12.7 × 8.4 cm, right 6.9 × 4.8 cm).

Ultrasound on April 3, 2024 demonstrated multiple hypoechoic nodules in both breasts and axillary regions (**Figure 1**). CT scan on April 15, 2024 revealed bilateral adnexal masses with diffuse pleural and peritoneal nodules, omental thickening, hepatic capsular lesions, and multiple enlarged lymph nodes (**Figure 2**).

**Figure 1:**
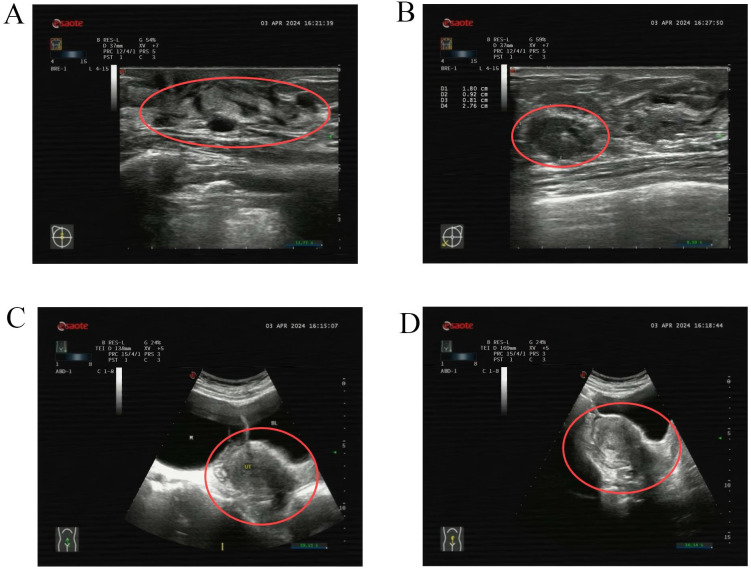
Initial breast and pelvic ultrasonography. **(A)** Right breast nodule measuring 11 × 6 mm. **(B)** Left breast nodule measuring 18 × 9 mm, classified as BI-RADS 4C. **(C, D)** Multiple irregular periuterine masses, with the largest measuring approximately 120 × 68 mm. Markers indicate representative lesions.

**Figure 2:**
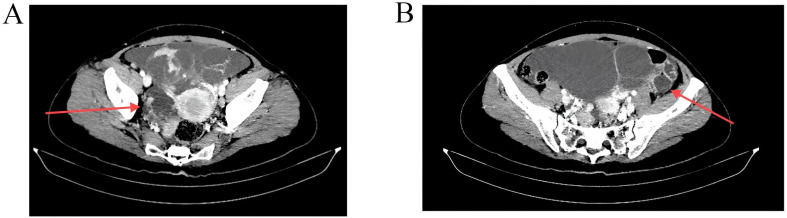
Initial pelvic computed tomography. **(A)** Right adnexal cystic-solid mass measuring 6.9 × 4.8 cm. **(B)** Left adnexal cystic-solid mass measuring 12.7 × 8.4 cm, suspicious for ovarian malignancy.

### Histopathology and immunohistochemistry

Pelvic biopsy on April 11, 2024 revealed high-grade serous adenocarcinoma with WT1+, PAX8+, CA125+, and Ki67 80% (**Figures 3A–D**). Left breast biopsy on the same day demonstrated two distinct components: primary breast invasive carcinoma (MG+, GATA3+, ER 5%, PR 50%, HER2 1+, Ki67 70%; **Figures 4A–D**) and metastatic Müllerian carcinoma (WT1+, PAX8+, CA125+). HE staining revealed two morphologically distinct adenocarcinoma components coexisting in the same lesion (**Figure 5**). Follow-up pelvic IHC confirmed ovarian origin of the metastatic component (**Figure 6**).

**Figure 3:**
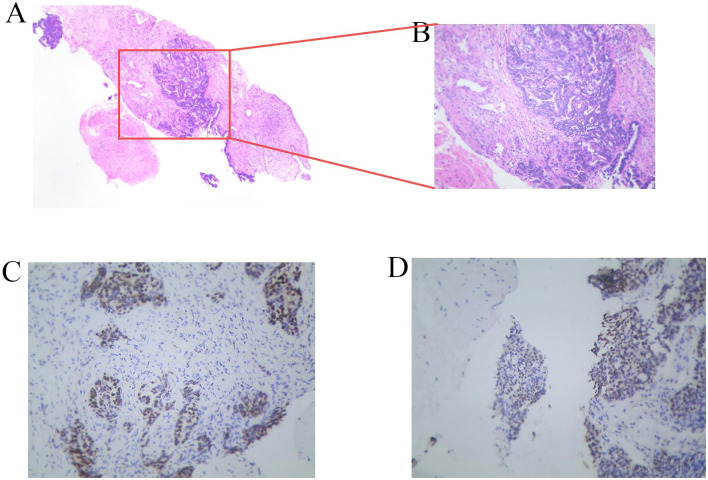
Pathological findings of the pelvic mass. **(A, B)** H&E staining showing high-grade serous carcinoma. **(C)** PAX8 nuclear positivity. **(D)** WT1 nuclear positivity, supporting Müllerian/ovarian origin.

**Figure 4:**
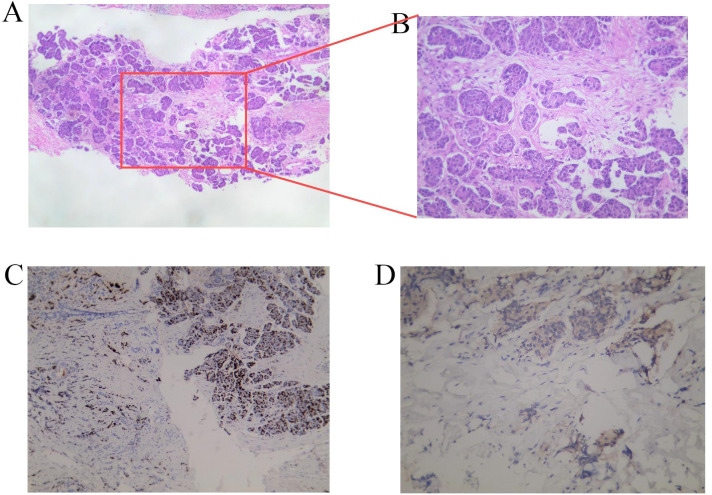
Pathological findings of the left breast lesion. **(A, B)** H&E staining showing invasive carcinoma with two morphologically distinct components. **(C)** High Ki67 expression. **(D)** HER2 weak membranous staining, scored as 1+.

**Figure 5:**
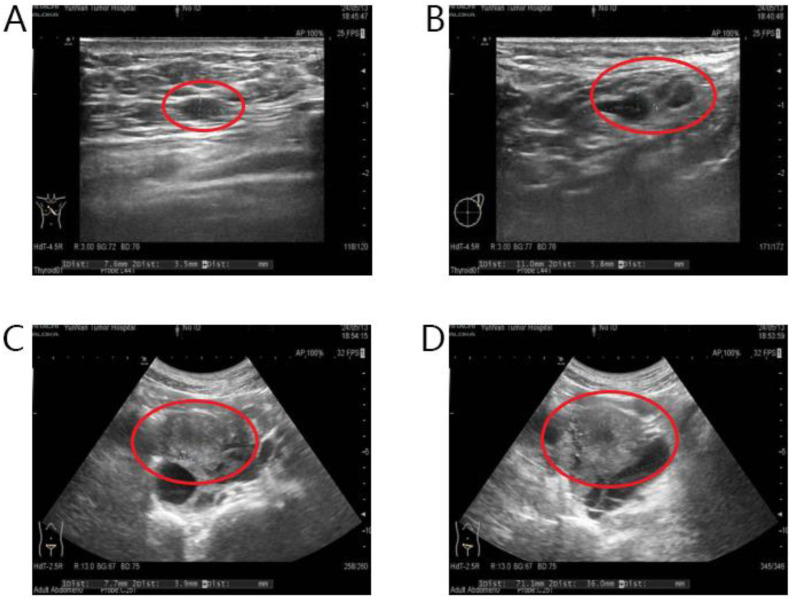
Ultrasound assessment after two cycles of paclitaxel plus carboplatin. Ultrasonography showed reduced bilateral breast lesions and pelvic masses after chemotherapy. Red circles indicate representative residual lesions. **(A)** Right breast. **(B)** Left breast. **(C, D)** Pelvic lesions.

**Figure 6:**
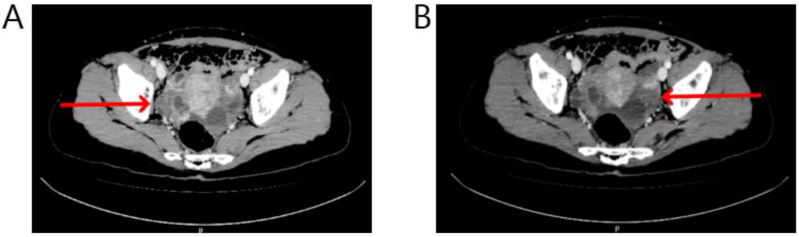
CT assessment after two cycles of paclitaxel plus carboplatin. **(A)** Right adnexal mass after treatment, decreased from 6.9 × 4.8 cm to 3.6 × 2.2 cm. **(B)** Left adnexal mass after treatment, decreased from 12.7 × 8.4 cm to 6.0 × 3.1 cm.

### Molecular testing

Molecular testing revealed a pathogenic BRCA mutation and homologous recombination deficiency (HRD)-positive status. These findings supported the use of PARP inhibitor maintenance therapy after platinum-based chemotherapy and suggested a possible homologous recombination repair-deficient background in this patient.

### Diagnosis

1. FIGO stage IVB high-grade serous ovarian carcinoma with pleural, peritoneal, lymph node, and breast involvement.
2. Synchronous primary invasive carcinoma of the left breast, grade 2, ER-low positive, PR-positive, HER2-negative, Ki67 high.
3. Bilateral breast lesions, with the left breast lesion showing two morphologically distinct components: primary breast invasive carcinoma and metastatic Müllerian/ovarian carcinoma.
4. Metastatic involvement of supraclavicular, axillary, thoracic, abdominal, retroperitoneal, and pelvic lymph nodes.

### Treatment timeline

1. Neoadjuvant chemotherapy: 2 cycles of TC (paclitaxel + carboplatin) in May 2024, resulting in partial response (PR).
2. Cytoreductive surgery: June 4, 2024 (pelvic tumor debulking).
3. Chemotherapy with TC + bevacizumab: 6 cycles between June and November 2024; bone marrow suppression managed symptomatically.
4. Left breast tumor excision: January 6, 2025; pathology confirmed stage IA primary breast carcinoma.
5. Maintenance therapy: oral fluzoparib (2 tablets BID) starting March 1, 2025, along with ongoing bevacizumab (17 cycles, last dose November 26, 2025).
6. Follow-up: December 20, 2025; no evidence of disease progression.

The major clinical events are summarized in **Table 1**.

**Table 1:** Clinical timeline of the patient.

Date	Clinical event	Key findings	Management
Feb 20, 2024	Admission to outside hospital	Encapsulated pleural effusion	Thoracoscopic pleurectomy and drainage
Feb 2024	Pleural biopsy	High-grade metastatic serous carcinoma	Further work-up for ovarian primary
Apr 1, 2024	Presentation to our institution	Abdominal distension and mild abdominal pain	Diagnostic evaluation
Apr 3, 2024	Ultrasound	Bilateral breast nodules, axillary lymphadenopathy, pelvic masses	BI-RADS 4C; ovarian malignancy suspected
Apr 11, 2024	Pelvic biopsy	High-grade serous carcinoma	IHC: WT1+, PAX8+, CA125+, Ki67 80%
Apr 15, 2024	CT	Bilateral adnexal masses, pleural/peritoneal/lymph node involvement	FIGO stage IVB considered
Apr 20, 2024	Chemotherapy	N/A	First cycle of TC (paclitaxel + carboplatin)
May 13, 2024	Chemotherapy/Ultrasound	Lesion reduction	Second cycle of TC (paclitaxel + carboplatin)/Response evaluation by ultrasound
May 31, 2024	CT follow-up	Partial response (PR); reduced bilateral adnexal masses and lymph nodes	N/A
Jun 4, 2024	Surgery	Pelvic tumor	Pelvic tumor debulking (cytoreductive surgery)
Jun–Nov 2024	Chemotherapy	Bilateral adnexal, breast, nodal lesions	Six cycles of TC + bevacizumab
Jan 6, 2025	Surgery	Left breast tumor	Left breast tumor excision
Mar–Nov, 2025	Maintenance therapy	N/A	Fluzoparib plus bevacizumab (targeted therapy)

HGSC, high-grade serous carcinoma; IHC, immunohistochemistry; TC, paclitaxel plus carboplatin; PR, partial response; CT, computed tomography.

## Literature review

### Search strategy and selection criteria

A focused literature search was conducted in PubMed to identify previously reported cases of ovarian, fallopian tube, or primary peritoneal carcinoma involving the breast and/or axillary lymph nodes. The search included all publications available from database inception to May 2026. The reference lists of relevant articles were also manually reviewed to identify additional eligible reports.

Articles were included if they met the following criteria: (1) the primary tumor was pathologically diagnosed as ovarian carcinoma, fallopian tube carcinoma, or primary peritoneal carcinoma; (2) breast involvement and/or axillary lymph node metastasis was clinically, radiologically, cytologically, or pathologically documented; and (3) sufficient clinicopathological information was available, including tumor histology, site of involvement, immunohistochemical findings, treatment, or clinical outcome. Articles were excluded if the reported breast lesion represented primary breast carcinoma without evidence of ovarian or Müllerian origin, if pathological or cytological confirmation was unavailable, or if essential clinical information was insufficient for analysis.

### Summary of previously reported cases

The available literature confirms that metastasis of ovarian or Müllerian-origin carcinoma to the breast region is rare. Reported cases most commonly involved serous carcinoma, particularly high-grade serous ovarian or primary peritoneal carcinoma. The breast and/or axillary lesions were identified either at the initial diagnosis of ovarian carcinoma or during recurrence after prior treatment. Clinically and radiologically, these lesions often resembled primary breast carcinoma or breast cancer-associated nodal disease, making pathological confirmation essential.

Immunohistochemistry plays a central role in determining tumor origin. Müllerian markers, including PAX8, WT1, and CA125, are commonly used to support ovarian, fallopian tube, or primary peritoneal origin. In contrast, breast-lineage markers, including GATA3, mammaglobin, GCDFP-15, ER, and PR, are useful for supporting primary breast origin. Because individual markers may show overlapping or focal expression, interpretation should be based on a combined panel together with tumor morphology, imaging findings, and clinical history.

Representative cases and reviews are summarized in **Table 2**. Most previously published reports described metastatic ovarian or primary peritoneal carcinoma involving the breast, axillary lymph nodes, or other supradiaphragmatic lymph nodes, without evidence of a synchronous primary breast carcinoma. In contrast, the present case is unusual because the breast biopsy revealed two morphologically distinct adenocarcinoma components: one compatible with primary breast invasive carcinoma and another morphologically discordant component showing Müllerian differentiation in the clinical context of advanced high-grade serous ovarian carcinoma. This coexistence creates a major diagnostic challenge and highlights the importance of integrating histopathology, immunohistochemistry, imaging, and clinical context when evaluating breast lesions in patients with ovarian carcinoma.

**Table 2:** Previously reported cases and reviews of ovarian or Müllerian-origin tumors involving the breast region and/or axillary or supradiaphragmatic lymph nodes.

Author, year	Age, years	Primary tumor	Timing of breast/axillary involvement	Site of involvement	IHC findings	Treatment	Outcome
Chamberlin et al., 2001 (32)	74	Serous borderline ovarian tumor	Internal mammary lymph node involvement was incidentally identified 7 years after excision of the ovarian tumor	Internal mammary lymph node; no breast parenchymal lesion reported	Metastatic tumor involving the internal mammary lymph node; detailed IHC findings were not available in the abstract	Initial excision of ovarian tumor; internal mammary lymph node detected during cardiac surgery	NR
Recine et al., 2004 (8)	21–67; 18 cases	Ovarian and/or primary peritoneal serous carcinoma	Breast and/or axillary lymph node involvement occurred in patients with ovarian/peritoneal serous carcinoma; most had advanced disease	Breast and/or axillary lymph nodes; 4 had multiple breast lesions, 8 had a single breast metastasis, and 6 had axillary lymph node involvement only	Papillary architecture and psammoma bodies were observed in some cases; WT-1 positivity and GCDFP-15 negativity in tested cases supported ovarian/peritoneal origin	NR	Follow-up available in 17 patients: 7 died of disease and 10 were alive with disease
Aydin et al., 2009 (33)	47	Stage IIIC serous ovarian carcinoma	Axillary lymph node metastasis occurred 32 months after cytoreductive surgery and adjuvant chemotherapy	Isolated axillary lymph node; no breast lesion reported	Histology of the axillary lymph node was consistent with serous ovarian carcinoma; IHC helped exclude breast origin	Cytoreductive surgery and adjuvant chemotherapy for ovarian cancer; management of axillary lesion	Tumor-free 12 months after axillary involvement
Ceccarelli et al., 2011 (34)	48	Ovarian carcinoma	Axillary lymph node metastasis was the initial presentation	Axillary lymph node; no breast parenchymal lesion reported	Fine-needle aspiration biopsy showed metastatic axillary lymphadenopathy; psammoma bodies were noted	NR	NR
Sibio et al., 2014 (35)	49	Serous papillary ovarian cancer with peritoneal carcinomatosis	Axillary lymph node enlargement was the first manifestation leading to diagnosis	Axillary lymph node; no breast lesion reported	Axillary lymph node biopsy showed metastasis consistent with ovarian origin	Peritonectomy and HIPEC followed by adjuvant systemic chemotherapy	Authors suggested that selected stage IV patients with isolated axillary metastasis may achieve outcomes comparable to stage IIIb–IIIc disease
Ueda et al., 2016 (36)	72	Stage III primary peritoneal serous carcinoma	Breast and axillary involvement occurred approximately 6 years after initial diagnosis and treatment	Breast and axillary lymph nodes	CK7(+), CK20(−), CA125(+), PAX8(+), GCDFP15(−), supporting metastatic peritoneal serous carcinoma rather than primary breast carcinoma	Initial paclitaxel plus carboplatin; breast-conserving surgery performed for suspected breast cancer	NR
Zuhdy et al., 2019 (37)	NR; 3 cases	One malignant mixed Müllerian tumor and two high-grade serous ovarian carcinomas	One synchronous case and two recurrent cases	Axillary lymph nodes; breast parenchymal metastasis not emphasized	IHC markers including WT-1, CK7, and CK20 were discussed as useful for distinguishing ovarian from breast origin	Individualized multidisciplinary management	NR/variable by case
Caruso et al., 2020 (38)	53	Advanced primary serous ovarian cancer	Breast and axillary metastases occurred 2 years after the diagnosis and treatment of ovarian cancer	Left breast and axillary lymph nodes	Pathology confirmed metastatic ovarian carcinoma involving the breast and axillary lymph nodes.	Primary cytoreductive surgery and platinum-based chemotherapy plus bevacizumab, followed by bevacizumab maintenance; after breast/axillary recurrence, axillary radiotherapy and multiple chemotherapy lines including gemcitabine/cisplatin, liposomal doxorubicin, topotecan, olaparib/cediranib, paclitaxel, and cisplatin	Died 3 years after disease recurrence
Koufopoulos et al., 2023 (39)	37–79; median 58; 25 cases	Ovarian carcinoma, predominantly serous carcinoma	13 cases presented with axillary lymph node metastasis at initial diagnosis; 12 developed axillary lymph node metastasis as recurrence	Isolated axillary lymph nodes; cases with breast, supraclavicular, or intramammary lymph node metastases were excluded	IHC panel including PAX8, WT1, p53, GATA3, and mammaglobin was recommended to distinguish ovarian from breast origin	Various treatments, including surgery, chemotherapy, radiotherapy, or combined approaches	No significant survival difference between initial-presentation and recurrent groups; CA-125 differed significantly between groups
Abdullah et al., 2024 (40)	54; 58	Case 1: grade 2 ovarian endometrioid carcinoma; Case 2: high-grade serous ovarian carcinoma	Axillary lymphadenopathy was present at initial diagnosis	Axillary lymph nodes; no breast involvement reported	Axillary lymph node biopsy showed metastatic adenocarcinoma; IHC supported ovarian origin and excluded breast origin	Surgery and chemotherapy	Both patients died within 3 years after diagnosis
Das and Ray, 2025/2026 (41)	54	Stage IVB high-grade serous ovarian carcinoma	Right axillary lymph node metastasis occurred during follow-up, approximately 3 years after initial treatment	Right axillary lymph node; no breast involvement on mammography	Axillary node biopsy confirmed metastatic high-grade serous ovarian carcinoma; PAX8(+) and WT1(+)	Second-line chemotherapy followed by secondary cytoreductive surgery, HIPEC with cisplatin, and right level I axillary lymph node dissection	Favorable local result; long-term outcome NR

IHC, immunohistochemistry; NR, not reported; HIPEC, hyperthermic intraperitoneal chemotherapy; HGSC, high-grade serous carcinoma; CK, cytokeratin; WT-1, Wilms tumor 1; PAX8, paired box gene 8; GCDFP-15, gross cystic disease fluid protein 15.

## Discussion

The present case represents a rare and diagnostically challenging scenario in which primary breast invasive carcinoma coexisted with a morphologically distinct Müllerian-type carcinoma component in the breast in a patient with advanced high-grade serous ovarian carcinoma. As summarized in our literature review, metastasis of ovarian or primary peritoneal serous carcinoma to the breast and/or axillary lymph nodes has been reported only sporadically. These lesions may closely mimic primary breast carcinoma on clinical examination and imaging, leading to potential diagnostic pitfalls. The current case further expands this diagnostic spectrum because the breast biopsy revealed two distinct adenocarcinoma components, one compatible with primary breast invasive carcinoma and the other showing features suggestive of metastatic Müllerian/ovarian origin. This finding highlights the importance of careful morphological assessment combined with an appropriate immunohistochemical panel when evaluating breast lesions in patients with ovarian carcinoma.

Breast cancer is the most commonly diagnosed malignancy among women worldwide and remains a major cause of cancer-related mortality (1). Although most malignant breast lesions represent primary breast carcinoma, metastatic tumors to the breast from extramammary malignancies are uncommon, accounting for less than 1% of all breast malignancies when contralateral breast cancer and lymphoma are excluded (2, 7). Among gynecologic malignancies, ovarian carcinoma metastasis to the breast or axillary lymph nodes is particularly rare, but it represents an important diagnostic pitfall because it may clinically and radiologically mimic primary breast carcinoma (8). Accurate distinction between primary breast cancer and metastatic ovarian carcinoma is essential, as these entities differ substantially in therapeutic strategy, surgical decision-making, and prognosis (7, 8).

High-grade serous ovarian carcinoma (HGSC) is the most common and aggressive histological subtype of epithelial ovarian cancer, which accounts for approximately 70%–80% of epithelial ovarian cancers (9–11). Globally, ovarian cancer was responsible for 3.7% of new cancer cases and 4.7% of cancer-related deaths among women in 2020 (12), with the majority of patients diagnosed at an advanced stage when peritoneal dissemination or distant metastasis has already occurred (12). HGSC is characterized by rapid progression, marked genomic instability, and a strong propensity for transcoelomic, lymphatic, and hematogenous spread, most frequently to the liver, lymph nodes, lung, bone, and brain (5). In contrast, metastasis to the breast is exceedingly rare, representing less than 0.6% of malignant breast tumors (6).

Several risk factors have been associated with HGSC, including age over 60 years, a family history of breast or ovarian cancer, and infertility, whereas multiparity, breastfeeding, oral contraceptive use, late menarche, and early menopause are considered protective (13, 14). Notably, some of these reproductive and hormonal factors also influence breast cancer risk, reflecting shared etiologic pathways between ovarian and breast malignancies. Approximately 20% of ovarian cancers are hereditary, predominantly linked to germline mutations in BRCA1 and BRCA2 genes (15). BRCA1 mutation carriers have a lifetime ovarian cancer risk of 44–61%, whereas BRCA2 carriers have a risk of up to 24% (15). These mutations also confer familial risk of breast cancer, accounting for approximately 5–10% of cases, with cumulative risk by age 70 reaching ~65% for BRCA1 and ~47% for BRCA2 carriers (16, 17). Identification of these hereditary risk factors is clinically important, as it informs genetic counseling, surveillance strategies, and preventive interventions such as prophylactic salpingo-oophorectomy.

Together, these epidemiologic and genetic characteristics underscore why HGSC frequently presents at an advanced stage with widespread dissemination, and why rare metastatic sites—such as the breast and axillary lymph nodes—can occasionally be involved, creating diagnostic challenges when breast lesions are evaluated in patients with a history or suspicion of ovarian carcinoma.

Genetically driven pathogenesis along an “ovarian–breast axis” may underlie both primary and metastatic processes involving the breast. High-grade serous ovarian carcinoma (HGSC) exhibits pervasive TP53 alterations, present in approximately 96% of cases according to TCGA data, while BRCA1/2 germline mutations occur in roughly 9% of patients (18–20). These shared genetic aberrations suggest overlapping molecular mechanisms between HGSC and breast neoplasia, emphasizing the clinical relevance of TP53- and BRCA-driven pathways.

Metastasis of ovarian carcinoma to the breast is exceedingly rare, typically occurring within two years of initial ovarian cancer diagnosis, with more than 60% of cases involving axillary lymph nodes (8, 21). Compared with primary breast cancers, metastatic breast tumors (MMTB), excluding contralateral breast tumors and lymphoma, remain uncommon (<1% of all breast malignancies) (22). Clinically and radiologically, MMTB often present as painless, firm nodules with well-circumscribed margins, occasionally bilateral, and usually lack typical features of primary breast carcinoma such as skin retraction or microcalcifications. Ultrasound typically demonstrates round or oval hypoechoic masses with clear borders. In our patient, multiple bilateral hypoechoic nodules in the breasts and axillary regions were consistent with these characteristics (23).

Differentiating primary from metastatic breast lesions is challenging, as imaging alone is often insufficient. Histopathological examination (HE staining) combined with immunohistochemistry is essential for definitive diagnosis. Markers including mammaglobin (MG)+, GATA3+, CK7/CK20, GCDFP-15/ER/PR support primary breast origin, whereas WT1+ and PAX8+ indicate Müllerian or ovarian origin (24–27). In the present case, left breast biopsy demonstrated a mixed immunophenotype with MG+/GATA3+/WT1+/PAX8+ (Ki67+, 70%), while pelvic biopsy showed WT1+/PAX8+ (Ki67+, 80%). HE staining revealed two morphologically distinct components: invasive ductal carcinoma coexisting with serous adenocarcinoma of ovarian or uterine origin, highlighting a rare scenario of synchronous primary and metastatic tumors within the breast.

Treatment strategies for HGSC and metastatic breast involvement depend on disease stage and patient condition. Surgery remains central for early-stage disease, while advanced cases often require neoadjuvant chemotherapy followed by interval cytoreductive surgery upon achieving sufficient tumor shrinkage. Maintenance targeted therapies, particularly PARP inhibitors, have demonstrated benefit in patients with BRCA-mutated tumors, according to NCCN guidelines (28). Surgical management addressing both ovarian primaries and metastatic breast lesions has been associated with improved survival; conversely, patients who do not undergo surgery face up to an 88% higher risk of mortality from metastatic solid tumors involving the breast (29–31).

In this case, the patient was diagnosed with FIGO stage IVB high-grade serous ovarian carcinoma involving the ovaries, breast, lymph nodes, pleura, and peritoneum. She achieved a partial response after neoadjuvant paclitaxel plus carboplatin chemotherapy and subsequently underwent interval debulking surgery. Molecular testing revealed a pathogenic BRCA mutation and HRD-positive status, which provided a molecular rationale for PARP inhibitor maintenance therapy. The germline or somatic origin of the BRCA mutation should be interpreted according to the original molecular testing report. If a germline BRCA mutation was confirmed or suspected, genetic counseling and cascade testing for family members should be recommended.

Overall, this case highlights a rare diagnostic scenario in which primary breast invasive carcinoma coexisted with metastatic high-grade serous ovarian carcinoma involving the breast. In patients with ovarian carcinoma who present with breast lesions, metastatic ovarian carcinoma should be considered in the differential diagnosis, even when a synchronous primary breast carcinoma is present. Comprehensive evaluation integrating clinical findings, imaging, morphology, immunohistochemistry, and molecular testing is essential for accurate diagnosis and individualized treatment. Molecular testing for BRCA mutations and HRD status may further guide maintenance therapy and genetic counseling.

## Data Availability

The original contributions presented in the study are included in the article/supplementary material. Further inquiries can be directed to the corresponding authors.
